# Multi-Sensor Image Fusion Method for Defect Detection in Powder Bed Fusion

**DOI:** 10.3390/s22208023

**Published:** 2022-10-20

**Authors:** Xing Peng, Lingbao Kong, Wei Han, Shixiang Wang

**Affiliations:** 1 Shanghai Engineering Research Center of Ultra-Precision Optical Manufacturing, Fudan University, Shanghai 200433, China; 2 College of Intelligence Science, National University of Defense Technology, Changsha 410073, China; 3 Key Laboratory of Science and Technology on Integrated Logistics Support, National University of Defense Technology, Changsha 410073, China; 4 Yiwu Research Institute, Fudan University, Chengbei Road, Yiwu City 322000, China

**Keywords:** powder bed fusion, multi-sensor image fusion, defect detection, visible imaging, infrared imaging

## Abstract

Multi-sensor defect detection technology is a research hotspot for monitoring the powder bed fusion (PBF) processes, of which the quality of the captured defect images and the detection capability is the vital issue. Thus, in this study, we utilize visible information as well as infrared imaging to detect the defects in PBF parts that conventional optical inspection technologies cannot easily detect. A multi-source image acquisition system was designed to simultaneously acquire brightness intensity and infrared intensity. Then, a multi-sensor image fusion method based on finite discrete shearlet transform (FDST), multi-scale sequential toggle operator (MSSTO), and an improved pulse-coupled neural networks (PCNN) framework were proposed to fuse information in the visible and infrared spectra to detect defects in challenging conditions. The image fusion performance of the proposed method was evaluated with different indices and compared with other fusion algorithms. The experimental results show that the proposed method achieves satisfactory performance in terms of the averaged information entropy, average gradient, spatial frequency, standard deviation, peak signal-to-noise ratio, and structural similarity, which are 7.979, 0.0405, 29.836, 76.454, 20.078 and 0.748, respectively. Furthermore, the comparison experiments indicate that the proposed method can effectively improve image contrast and richness, enhance the display of image edge contour and texture information, and also retain and fuse the main information in the source image. The research provides a potential solution for defect information fusion and characterization analysis in multi-sensor detection systems in the PBF process.

## 1. Introduction

Powder bed fusion (PBF) is a mature additive manufacturing (AM) technology, and its procedure mainly includes material supply, preparation, processing, and post-processing [1]. During PBF processes, the 3D model of the part is cut into thin layers, the metal powders are spread into the powder bed and reservoir by a recoating mechanism, and the machining system utilizes a high-power density laser to selectively melt the metal layer-by-layer [2]. The PBF can efficiently manufacture complex 3D structures [3] and carry out in situ alloying treatment [4,5]. During processing, the laser spot with Gaussian energy distribution interacts with the powder, and the powder particles melt and solidify instantaneously, resulting in many complex non-equilibrium chemical and physical metallurgical processes involving fluid flow, momentum, and mass and heat transfer, etc. [6,7]. Presently, various alloys and metals have been successfully processed using PBF technology, including aluminum alloys [8], stainless steels [9], nickel-based superalloys [10], and titanium alloys [11]. In the PBF process, many factors affect the quality of parts, including powder size, laser power, scanning speed, etc. Improper parameter control would lead to defects and seriously deteriorate the physical and mechanical properties of the parts [12]. However, the PBF parts still have major limitations in the production process for two main reasons: quality and repeatability, which may be seriously affected by certain defects (such as porosity, spheroidization, etc.) [13]. Many existing defect detection technologies rely on visible imaging sensors. For example, Joschka et al. [14] proposed a high-resolution defect detection system to detect topological defects and the surface quality of powder deposition layers. Grasso et al. [15] studied a method of defect space recognition and detection using a visible light camera in the layered process. Caltanissetta et al. [16] proposed using a measurement system to characterize the accuracy of original contour recognition in PBF layered images. Zheng et al. [17] proposed a visual detection system based on the extraction of the plume, molten pool, and splash features in the processing process. Due to the influence of lighting and the processing environment, the brightness of the defect detection image would be uneven and the richness of information would be low, which would affect the detection accuracy. It is difficult for visible light imaging sensors to provide higher imaging quality to distinguish various types of defects. To solve this problem, much research on PBF defect detection technology has been studied to improve the processing quality of parts, among which a series of detection systems composed of multi-sensors have emerged, namely multi-sensor detection systems [12], which are based on multi-sensor detection of light, sound, heat, and other signals providing more comprehensive, reliable, and accurate information for defect detection and characterization in the PBF process [13,18,19,20,21,22,23,24,25].

Yakout et al. [25] proposed an in situ detection system consisting of a high-speed infrared thermal camera and an infrared pyrometer to detect powder delamination and spattering in the SLM processes. Gusarov et al. [23] developed a detection system consisting of a high-speed CCD camera, a near-infrared camera, and a pyrometer to diagnose the SLM process under different laser power densities and obtained the relationship between geometric parameters of each machining trajectory and the laser power density distributions. Craeghs et al. [18], Tatsuaki et al. [19], and Sebastian et al. [20] investigated continuous detection of high-speed melt pools in SLM processes to achieve real-time feedback control of process parameters. The in situ detection system is mainly composed of a CCD (charge coupled device)/CMOS (complementary metal oxide semiconductor) camera, a photodiode, and a data acquisition and processing system. Aniruddha et al. [24] studied SLM process data over a wide range of laser velocities and laser powers using a high-speed camera and a pyrometer. Gould et al. [21] proposed a detection method combining high-speed infrared imaging with high-speed X-ray imaging to detect the vapor plume flow mechanics, cooling rate, splash, and molten pool three-dimensional morphology. However, when using the multi-sensor system to detect and characterize the defects of PBF parts, the quality of the detection images captured by the sensors is likely to be poor due to uncertain factors such as changes in the detection environment, resulting in difficulty distinguishing defect detail features. However, enhancing image quality only by improving hardware is not only difficult and time-consuming, but also costly. Therefore, the research on multi-sensor image data fusion technology is quite significant.

In the field of visual inspection, visible light imaging can provide detailed information, which is conducive to improving detection capabilities and ensuring detection accuracy. However, its imaging quality is seriously affected by the light environment, and it is difficult to detect defects such as powder coverage and strongly reflected light annihilation. Infrared imaging has good penetrative ability and thermal contrast and is less affected by complex environments such as powder splashing, but it is difficult to capture defect details and has low detection accuracy. Therefore, designing a multi-sensor system to capture and fuse the visible light and infrared information is significant for defect detection in PBF processes. The fusion image of infrared and visible images has the advantages of good target recognition, high spatial resolution, rich background details, etc., which can effectively improve the correct detection probability and target positioning ability in a complex environment. Through reasonable image fusion method design, the multi-sensor detection system can capture defect information clearly and accurately, and effectively realize defect feature extraction and analysis.

Image fusion technology is a key technology to fuse complementary and redundant information in multiple images of the same scene into a high-quality fusion image. The image information after fusion processing is rich, which is convenient for subsequent defect identification and characterization. Presently, image fusion technology has been widely used in many industrial fields such as computer vision, medical imaging, remote sensing, security, and monitoring [26]. Generally, image fusion is divided into three levels: pixel level, feature level, and decision level. In pixel-level image fusion, more attention is paid to the information expression of each pixel in the fused image, which can retain as much information about the source image as possible. The data fusion method discussed in this paper is pixel-level fusion. Pixel-level image fusion can be divided into spatial domain fusion and transform domain fusion. Spatial fusion methods use typical pixels of source images to construct fused images, such as independent component analysis [27], principal component analysis (PCA) [28], sparse representation (SR) [29], etc. However, in the spatial domain fusion method, the source image information is insufficiently utilized, and the phenomenon of blurring effect and contrast reduction can easily occur and transform domain fusion can be an effective method to solve this problem. Transform domain fusion is complete information fusion in the transform domain on the assumption that some features can be more textured in the transform domain. It consists of three steps: (1) decompose the source image into multi-scale sub-bands; (2) fuse partial sub-bands; (3) reconstruct the fused image from the fused sub-bands. For transform domain fusion methods, choosing a multi-scale decomposition tool and designing a sub-band image fusion strategy are two key issues.

In the research of multi-scale decomposition tools, many fusion methods have been proposed, such as pyramid transform [30], contourlet transform (CT) [31], non-subsampled contourlet transform (NSCT) [32], discrete wavelet transform (DWT) [33], shift-invariant discrete wavelet transform (SIDWT) [34] and so on. Finite discrete shearlet transform (FDST) can obtain more direction selectivity and faster computation speed than traditional discrete wavelet transforms [35]. However, when the FDST is used to fuse images with large differences in grayscale features, the shortcomings of poor contrast and unclear details are prone to occur. The multi-scale sequential toggle operator (MSSTO) can extract the bright and dark features of the source image and then fuse them with the source image, which can effectively improve the image contrast [36]. Pulse-coupled neural networks (PCNN) are single-layer neural network mathematical models established by interconnecting countless neurons through link coefficients. Thanks to the pulse synchronization and global coupling of neurons, it can make full use of local pixel information, suitable for designing fusion rules and determining fusion coefficients [37]. The PCNN can effectively overcome the shortcomings of the above-mentioned high-frequency coefficient fusion methods.

In this paper, an image fusion algorithm based on FDST-MSSTO and improved PCNN is proposed, named FMP. The process of the FMP method roughly includes: using FDST to decompose the low-frequency sub-band coefficients and high-frequency sub-band coefficients of the source image; using MSSTO to extract the image detail bright information in the low-frequency sub-band coefficients and dark information; fusing the extracted light and dark information and low-frequency coefficients to obtain low-frequency fusion coefficients; using the improved MSF-PCNN method to obtain high-frequency fusion coefficients; reconstructing the fusion image through FDST inverse transform. The FMP method can effectively improve image contrast and information richness and improve the display of image edge contour and texture information, which is of great significance for the fusion and analysis of defect information in multi-sensor detection systems. This paper is organized as follows. The FMP image fusion method is described in Section 2. In Section 3, the experimental studies and discussion are presented. Section 4 describes the conclusions.

## 2. FMP Image Fusion Method

### 2.1. FDST

The parabolic scaling $A_{a}$ and shearing matrices $S_{s}$ are:

(1) $$A_{a}=\left[\begin{array}{cc} a & 0\\ 0 & \sqrt{a} \end{array}\right],S_{s}=\left[\begin{array}{cc} 1 & s\\ 0 & 1 \end{array}\right],s\in R$$

Function $\psi \in L^{2}\left(R^{2}\right)$ through expansion, shearing, and translation:

(2) $$\psi_{a,s,t}\left(x\right)=a^{-\frac{3}{4}}\psi \left(A_{a}^{-1}S_{s}^{-1}\right)\left(x-t\right)$$

where *a* is the scale parameter, *s* is the shear parameter, and *t* is the translation parameter.

Then, a two-dimensional Fourier transform is performed on the function $\psi_{a,s,t}\left(x\right)$ to obtain the continuous shearlet transform of any function in the $L^{2}\left(R^{2}\right)$ and the corresponding Parseval equation as follows:

(3) $$\{\begin{array}{l} SH_{\psi }\left(f\right)=<f,\psi_{a,s,t}>=<\hat{f},{\hat{\psi }}_{a,s,t}>\\ \hat{f}\left(w\right)=\int_{R^{2}}f\left(t\right)e^{-2\pi i<\omega ,t>}dt\\ {\hat{\psi }}_{a,s,t}\left(w\right)=a^{\frac{3}{4}}e^{-2\pi i<\omega ,t>}\hat{\psi }\left(aw_{1},\sqrt{a}\left(sw_{1}+w_{2}\right)\right) \end{array}$$

Meanwhile, the wavelet function ${\hat{\psi }}_{1}\left(w_{1}\right)$ and impulse function ${\hat{\psi }}_{2}\left(w_{2}\right)$ are defined as:

(4) $${\hat{\psi }}_{1}\left(w_{1}\right)=\sqrt{b^{2}(2w_{1})+b^{2}(w_{1})}$$

(5) $${\hat{\psi }}_{2}\left(w_{2}\right)=\{\begin{array}{l} \sqrt{v(1+w_{2})},w_{2}\leq 0\\ \sqrt{v(1-w_{2})},w_{2}>0 \end{array}$$

**Figure 1:**
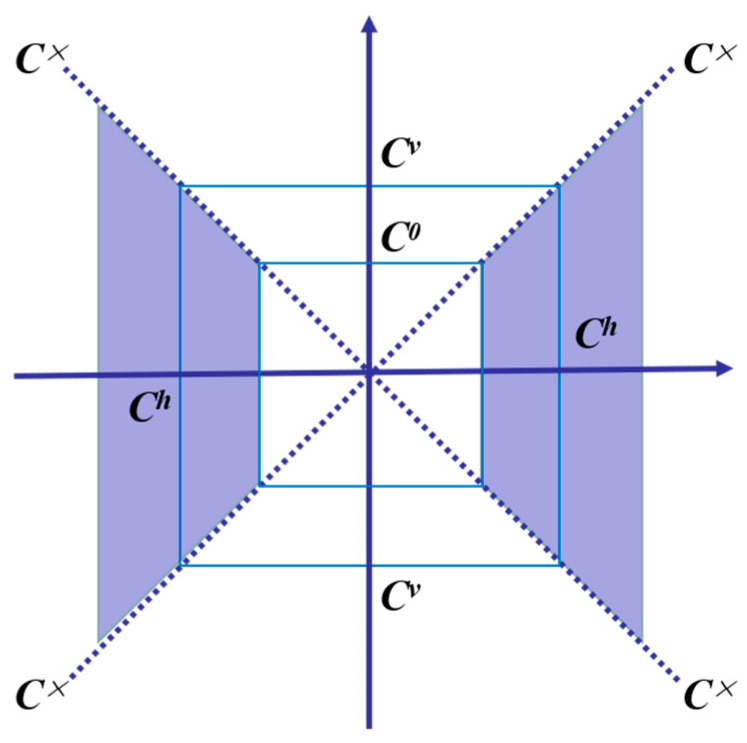
Schematic diagram of frequency domain plane division.

Then, the wavelet function ${\hat{\psi }}_{1}\left(w_{1}\right)$ and impulse function ${\hat{\psi }}_{2}\left(w_{2}\right)$ is used to decompose the frequency domain into four parts: horizontal cone *C^h^*, vertical cone *C^v^*, cross line of cone *C*^×^, and low-frequency *C*^0^ [34]. The decomposition method is shown in Figure 1.

Based on the continuous shear wave function $\psi_{a,s,t}\left(x\right)$, the shear parameters, scale parameters, and translation parameters in Equation (2) are discretized, and then the discrete shear wave transform is obtained from the continuous shear wave transform, obtaining $\psi_{j,k,m}^{h}$ and $\psi_{j,k,m}^{v}$ in the region *C^h^* and *C^v^*. At the boundary of the cone $\psi_{j,k,m}^{h\times v}=\psi_{j,k,m}^{v}+\psi_{j,k,m}^{h}+\psi_{j,k,m}^{\times }$, the discrete shear wave transform can be defined as:

(6) $$SH\left(f\right)\left(\kappa ,j,k,m\right)=\{\begin{array}{l} <f,\phi_{m}>,\kappa =0\\ <f,\psi_{j,k,m}^{\kappa }>,\kappa \in \left\{h,v\right\}\\ <f,\psi_{j,k,m}^{h\times v}>,\kappa =\times \end{array}$$

The discrete shearlet transform defined by Equation (6) can be realized by a two-dimensional fast Fourier transform, which has low computational complexity and good multi-scale decomposition characteristics and can realize the low-frequency sub-band information of the image, and the decomposition of high-frequency sub-band information.

### 2.2. MSSTO

Mathematical morphology is widely used in image processing [38]. Suppose $f_{l}^{X}\left(x,y\right)$ and C(u,v) represent a collection of source images and structuring elements, respectively. (x,y) represents the coordinates of the pixels in the source image and (u,v) represents the coordinates of the pixels in the structuring element *C.* Dilation and erosion operations are defined with $f_{l}^{X}\left(x,y\right)$ and C(u,v), expressed as follows:

(7) $$\{\begin{array}{c} f_{l}^{X}⊕C=\max\left(f_{l}^{X}\left(x-u,y-v\right)+C\left(u,v\right)\right)\\ f_{l}^{X}⊖C=\min\left(f_{l}^{X}\left(x+u,y+v\right)-C\left(u,v\right)\right) \end{array}$$

where ⊕ and ⊖ represent the operations of dilation and corrosion, respectively.

By combining dilation and erosion, the opening and closing operations are defined as follows:

(8) $$\{\begin{array}{c} f_{l}^{X}\circ C=(f_{l}^{X}⊖C)⊕C\\ f_{l}^{X}•C=(f_{l}^{X}⊕C)⊖C \end{array}$$

where ∘ represents the opening operation, and • represents the closing operation. The opening operation and closing operation can effectively smooth the bright and dark features of the image. Based on the above opening and closing operations, the one-time flip operator (TO) is defined as [36]:

(9) $$TO\left(f_{l}^{X}\right)\left(x,y\right)=\{\begin{array}{l} f_{l}^{X}\circ C\left(x,y\right),if\;f_{l}^{X}•C\left(x,y\right)-f_{l}^{X}<f_{l}^{X}-f_{l}^{X}C\left(x,y\right)\\ \begin{array}{l} f_{l}^{X}•C\left(x,y\right),if\;f_{l}^{X}•C\left(x,y\right)-f_{l}^{X}>f_{l}^{X}-f_{l}^{X}C\left(x,y\right)\\ f_{l}^{X},\mathrm{else} \end{array} \end{array}$$

where $f_{l}^{X}\left(X=A,\;B\right)$ is the low-frequency sub-band coefficients of source image *A* and source image *B.* Image features usually exist on multiple scales of images and extracting these multi-scale image features is key to image fusion. Therefore, the multi-scale continuous flip operator is defined by using multi-scale structuring elements:

(10) $$\{\begin{array}{l} STO_{C_{i}}\left(f_{l}^{X}\right)=TO_{C_{i}}\left(STO_{C_{i-1}}\left(f_{l}^{X}\right)\right)\\ STO_{C_{1}}\left(f_{l}^{X}\right)=TO_{C_{1}}(f_{l}^{X})\\ STO_{0}\left(f_{l}^{X}\right)=f_{l}^{X} \end{array}$$

where $C_{i}$ is the structuring element on the scale i.

The light information and dark information of source image *A* and source image *B* are fused, respectively by using a weighting strategy, which is expressed as:

(11) $$\{\begin{array}{l} BFF_{C_{i}}\left(x,y\right)=mA_{C_{i}}\times BF_{C_{i}}\left(f_{l}^{A}\right)\left(x,y\right)+mB_{C_{i}}\times BF_{C_{i}}\left(f_{l}^{B}\right)\left(x,y\right)\\ BF_{C_{i}}\left(f_{l}^{X}\right)\left(x,y\right)=\max\left(STO_{C_{i-1}}\left(f_{l}^{X}\right)\left(x,y\right)-STO_{C_{i}}\left(f_{l}^{X}\right)\left(x,y\right),0\right),X=A,B\\ DFF_{C_{i}}\left(x,y\right)=nA_{C_{i}}\times DF_{C_{i}}\left(f_{l}^{A}\right)\left(x,y\right)+nB_{C_{i}}\times DF_{C_{i}}\left(f_{l}^{B}\right)\left(x,y\right)\\ DF_{C_{i}}\left(f_{l}^{X}\right)\left(x,y\right)=\max\left(STO_{C_{i}}\left(f_{l}^{X}\right)\left(x,y\right)-STO_{C_{i-1}}\left(f_{l}^{X}\right)\left(x,y\right),0\right),X=A,B \end{array}$$

where $BF_{C_{i}}\left(f_{l}^{X}\right)\left(x,y\right)$ represents the bright detailed information of the image’s low-frequency sub-band coefficients $f_{l}^{X}\left(X=A,\;B\right)$ at the scale i, $DF_{C_{i}}\left(f_{l}^{X}\right)\left(x,y\right)$ represents the dark detail information of the image’s low-frequency sub-band coefficients $f_{l}^{X}\left(X=A,\;B\right)$ at the scale i, $STO_{C_{i-1}}$ is the smooth image feature from scale i to scale i−1, $STO_{C_{i}}$ is the smooth image feature from scale 1 to i, $mA_{C_{i}}$ is the ratio of the mean value of bright information of the low-frequency coefficient of source image *A* on scale i to the sum of the mean values of bright information of source image *A* and source image *B*. $mB_{C_{i}}$ is the ratio of the mean value of bright information of the low-frequency coefficient of source image *B* on scale i to the sum of the mean values of bright information of source image *A* and image *B*. 

Finally, the bright fusion information and dark fusion information of the image are extracted by using the method of taking large pixel values of $BF_{C_{i}}\left(x,y\right)$ and $DF_{C_{i}}\left(x,y\right)$ as follows:

(12) $$\{\begin{array}{c} MBF\left(x,y\right)=\max\left(BF_{C_{i}}\left(x,y\right)\right)\\ MDF\left(x,y\right)=\max\left(DF_{C_{i}}\left(x,y\right)\right) \end{array}$$

The low-frequency sub-band image of the source image contains the main information of the source image [30], so choosing appropriate low-frequency sub-band coefficients can help to extract the key information of the image and improve the visual effect of the image. The fusion of the low-frequency coefficient (FLFC) strategy is to perform the MSSTO transformation on the low-frequency sub-band coefficients of source image *A* and source image *B* processed by the FDST to extract the bright and dark information of key features and combine them with the original image and fuse them with the low-frequency sub-band coefficients. The processed image features are smooth, and the edge details are rich, which can significantly improve the contrast of the image. The specific fusion strategy is expressed as:

(13) $$f_{l}^{F}\left(i,j\right)=\frac{\left[f_{l}^{A}\left(i,j\right)+f_{l}^{B}\left(i,j\right)\right]}{2}-\gamma \times MBF\left(i,j\right)+\varepsilon \times MDF\left(i,j\right)$$

where $f_{l}^{A}\left(i,j\right)$ is the low-frequency sub-band fusion coefficient at the position (i,j), γ and ε are the low-frequency sub-band fusion weight coefficients used to improve the contrast of the fused image.

### 2.3. Improved PCNN

**Figure 2:**
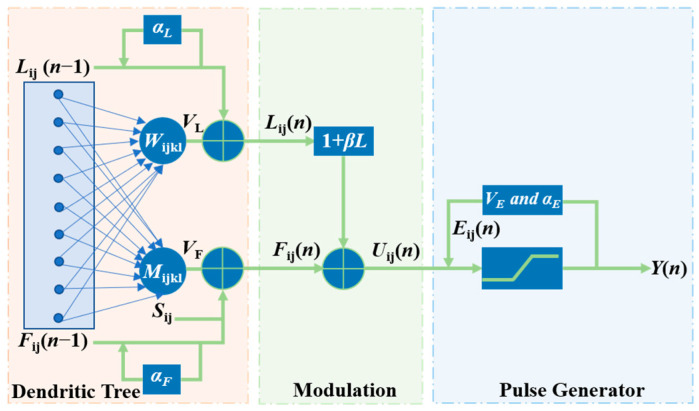
Schematic diagram of the neuron structure of PCNN.

PCNN is a two-dimensional feedback network for high-performance biomimetic image processing with nonlinear multiplication, linear addition, and coupled modulation characteristics, consisting of a branching tree, modulation domain, and pulse generator [39], as shown in Figure 2. The neurons in the PCNN correspond to the pixels of the image one by one, which can capture the subtle changes and detailed information of the image, maintain the integrity of the two-dimensional information of the input image, and combine the visual characteristics of the PCNN with the information characteristics of the image to improve the performance image fusion.

As shown in Figure 2, the input signal $S_{ij}$ is transformed into feedback input channel *F_ij_* and connection input channel *L_ij_* through the branching tree. In the modulation domain, the neuron internal activity term *U_ij_* combines the decaying feedback input *F_ij_* and the connecting input channel *L_ij_*. Finally, by comparing the internal activity term *U_ij_* with the dynamic threshold *E_ij_*, the neuron decides whether to generate a spike or not. The mathematical model of PCNN neuron discrete is expressed as Equations (14) and (15):

(14) $$\{\begin{array}{l} F_{ij}\left(n\right)=\exp\left(-\alpha_{F}\right)F_{ij}\left(n-1\right)+V_{F}\sum_{kl}M_{ijkl}Y_{kl}\left(n-1\right)\\ L_{ij}\left(n\right)=\exp\left(-\alpha_{L}\right)L_{ij}\left(n-1\right)+V_{L}\sum_{kl}M_{ijkl}Y_{kl}\left(n-1\right)\\ U_{ij}\left(n\right)=F_{ij}\left(n\right)(1+\beta_{ij}L_{ij}\left(n\right))\\ \begin{array}{l} E_{ij}\left(n\right)=\exp\left(-\alpha_{E}\right)E_{ij}\left(n-1\right)+V_{E}Y_{ij}\left(n\right)\\ T_{ij}\left(n\right)=T_{ij}\left(n-1\right)+Y_{ij}\left(n\right) \end{array} \end{array}$$

(15) $$Y_{ij}\left(n\right)=\{\begin{array}{l} 1,U_{ij}\left(n\right)>E_{ij}\left(n\right)\\ 0,\mathrm{otherwise} \end{array}$$

where *n* is the number of iterations, the subscript *ij* is the neuron label, $\alpha_{F}$, $\alpha_{E}$ and $\alpha_{L}$ are the attenuation coefficients, *M_ijkl_* links the weight matrix, *β_ij_* is the link strength, and *Y_ij_* is the output item.

The high-frequency sub-band image of the source image contains the edge and contour details of the image, etc. [34]. The traditional fusion of high-frequency coefficient (FHFC) method usually selects the fusion coefficient with a larger absolute value, but this method is easy to lose the image information and is sensitive to noise. Therefore, the FHFC strategy is to first calculate the MSF value of the high-frequency sub-band coefficient and use it as the external excitation of the PCNN. Compared with the traditional spatial frequency method, the gradient energy in the diagonal direction of the two images is calculated more, and more abundant image information can be extracted. For an image I with pixels of X×Y, the MSF value can be expressed as:

(16) $$\{\begin{array}{l} MSF=\sqrt{CF^{2}+RF^{2}+{\left(H+J\right)}^{2}}\\ RF=\sqrt{\frac{1}{X\left(Y-1\right)}}\sum_{x=1}^{X}\sum_{y=2}^{Y}{\left(I_{x,y}-I_{x,y-1}\right)}^{2}\\ CF=\sqrt{\frac{1}{\left(Y-1\right)X}}\sum_{x=2}^{X}\sum_{y=1}^{Y}{\left(I_{x,y}-I_{x-1,y}\right)}^{2}\\ \begin{array}{c} H=\sqrt{\frac{1}{\left(X-1\right)\left(Y-1\right)}}\sum_{x=2}^{X}\sum_{y=2}^{Y}{\left(I_{x-1,y}-I_{x,y-1}\right)}^{2}\\ J=\sqrt{\frac{1}{\left(X-1\right)\left(Y-1\right)}}\sum_{x=2}^{X}\sum_{y=2}^{Y}{\left(I_{x,y}-I_{x-1,y-1}\right)}^{2} \end{array} \end{array}$$

where CF is the gradient energy in the vertical direction, RF is the gradient energy in the horizontal direction, and H and J are the gradient energy in the diagonal direction.

## 3. Experimental Studies and Discussion

### 3.1. FMP Image Fusion Procedure

**Figure 3:**
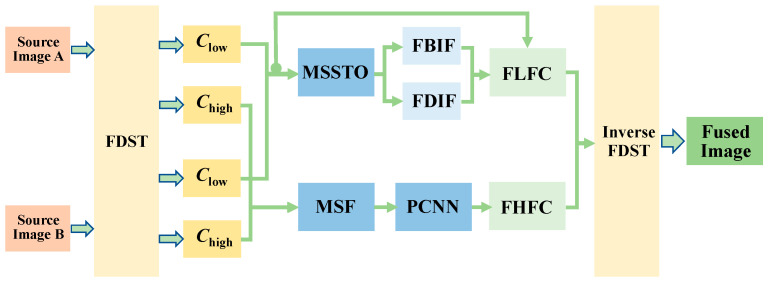
The schematic diagram of the proposed FMP: FBIF: fusion of bright feature; FDIF: fusion of dark feature; FHFC: fusion of high-frequency coefficient; FLFC: fusion of low-frequency coefficient; *C*_low_: low-frequency coefficient; *C*_high_: high-frequency coefficient.

Figure 3 shows the schematic diagram of the proposed FMP method. The procedure is as follows:

1. Using the FDST to decompose the registered image *A* and image *B* into low-frequency sub-band coefficients and high-frequency sub-band coefficients, respectively.
2. In the FDST transform domain, the MSSTO transform is used to extract the image detail bright and dark information in the low-frequency sub-band coefficients of image *A* and image *B*, respectively.
3. The light and dark information of the image extracted by MSSTO are merged into the low-frequency coefficients after fusion, and the low-frequency fusion coefficients are obtained.
4. In the FDST transform domain, the modified spatial frequency (MSF) is used to extract the gradient energy of the image in the vertical, horizontal, and diagonal directions, and the high-frequency sub-band coefficient MSF value is calculated, which is used as the external excitation of the PCNN.
5. Using the PCNN criterion to obtain high-frequency fusion coefficients.
6. The final fused image is reconstructed from the fused low-frequency sub-band fusion coefficients and the high-frequency sub-band fusion coefficients using the FDST inverse transform.

### 3.2. Experimental Setup

**Figure 4:**
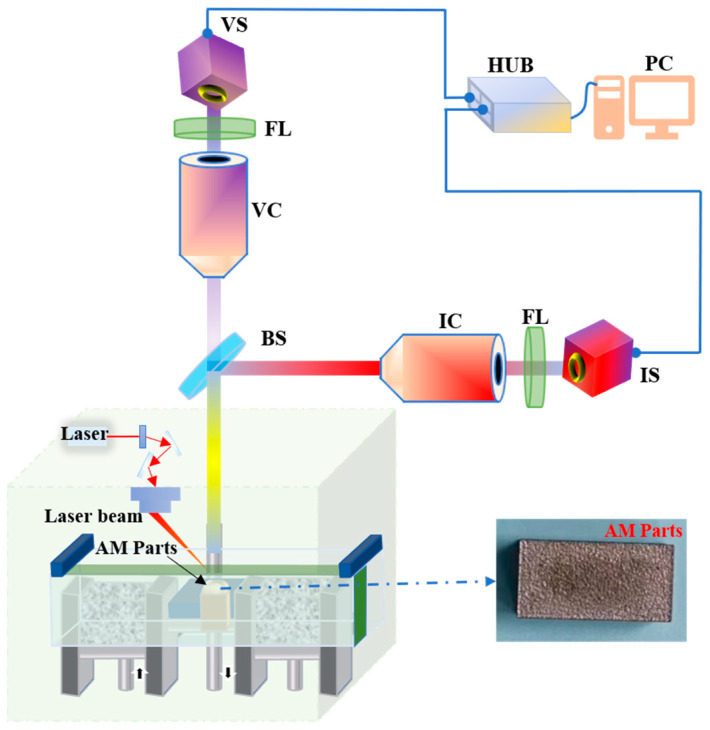
Schematic diagram of the experimental setup for defect detection in PBF processes: IC: Infrared channel imaging system; VC: Visible channel imaging system; BS: Beam-splitter; FL: Filters; IS: Infrared channel image sensor; VS: Visible channel image sensor; PC: computer.

To verify the effectiveness of the FMP method in improving the contrast of image fusion and extracting edge contour information, experimental analysis and evaluation were carried out. The experimental setup for defect detection in PBF processes mainly included the visible and infrared light systems, as shown in Figure 4. The design parameters of the defect detection system are listed in Table 1. The visible light system included a visible light imaging objective lens, a filter (GCC-301031, DAHENG OPTROELECTRONICS, Beijing, China), and a CMOS camera with a resolution of 7728 × 5368 and a single-pixel size of 1.1 μm, the focal length of the visible system is 50 mm, and the F-number is 2.5. The effective frame rate of the VS was 60 fps. The infrared system included an infrared imaging objective lens, a filter (NENIR03B, THORLABS, Shanghai, China), and an InGaAs sensor with a resolution of 320 × 256 and a single-pixel size of 30 μm, the focal length of the infrared system is 50 mm, and the F-number is 1.5. The effective frame rate of the IS was 25 fps, and the maximum allowable frame rate between the HUB and the PC was 25 fps. Additionally, the system also included an AM part, a beam splitter (BSW30, THORLABS, Shanghai, China), a hub, and an image-processing computer. The visible system worked within the wavelength band of 0.4–0.7 μm, and the infrared system within the near-infrared band of 0.9–1.7 μm. The image processing environment of the experiment was an Intel (R) Core (TM) i7-7700 CPU processor, a 64-bit Windows 10 version operating system with 16 G memory, and a MATLAB2021 software operating platform. In the experiment, we selected gradient pyramid (GP) [39], Laplacian pyramid (LP) [40], ratio of low-pass pyramid (RP) [41], PCA [27], dual-tree complex wavelet transforms (DT-CWT) [42], and PCNN [43] as the comparison objects. The performance comparison of the image fusion method was carried out through subjective evaluation and seven objective evaluation items.

### 3.3. Defect Detection and Discussion

**Figure 5:**
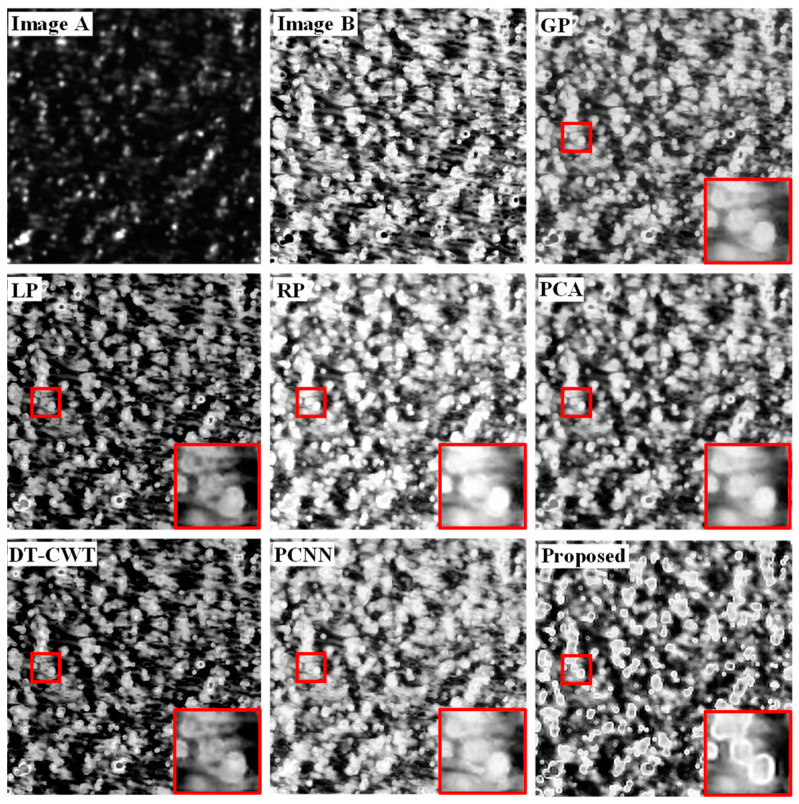
Fusion results of dataset 1 under different fusion algorithms.

**Figure 6:**
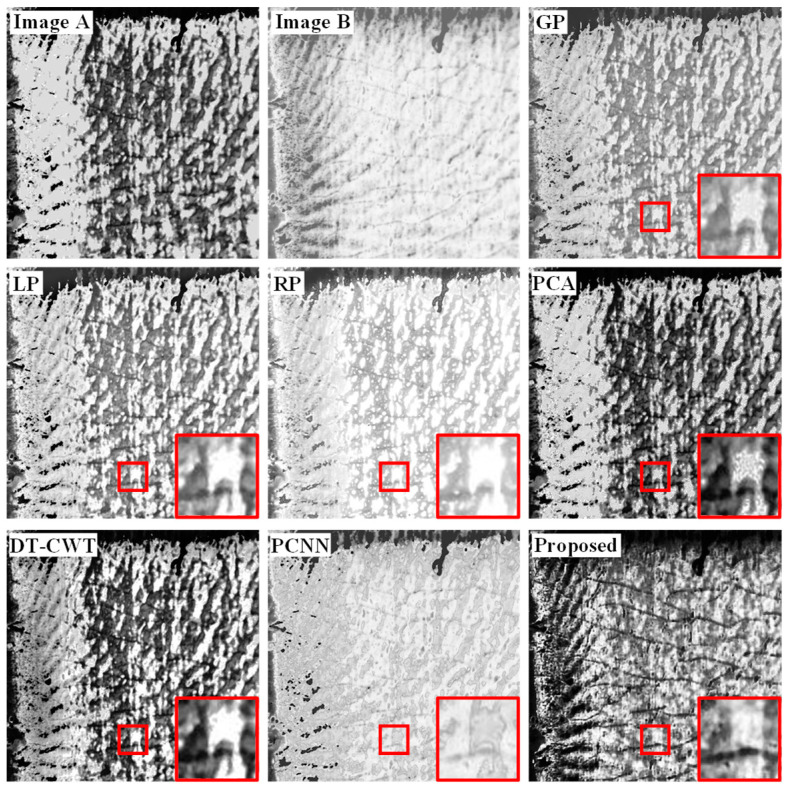
Fusion results of dataset 2 under different fusion algorithms.

Figure 5 and Figure 6 are the comparison of the image fusion results of dataset 1 and dataset 2, in which image *A* and image *B* are the infrared and visible defects detected images to be fused, all of them are from PBF parts. The area selected by the red box in Figure 5 and Figure 6 contains typical defects in laser AM workpiece, especially for the region of interest (ROI) to perform image fusion. Figure 5 indicates the common balling defects in PBF processes, while Figure 6 indicates the cracking defects. In the fusion result, the local content of the image is highlighted with a red frame for enlargement processing, and it is placed in the lower right corner of the image. These different algorithms can fuse the main information in image *A* and image *B* to varying degrees, and the visual effect is improved compared to an original single image. The overall effect of the image processed by the GP, PCA, DT-CWT, and PCNN algorithms is relatively blurred, and the contrast of the frame selection area is poor, which can easily cause the loss of key target information. The image contrast of the LP and RP algorithms has been improved, but the overall image after the LP processing is relatively dim, the edge retention of the target area of the image processed by the two algorithms is poor, and the contour texture features are not clear enough. As shown in Figure 5 and Figure 6, when the multi-sensor system is used for defect detection of laser AM parts, the defect detection images captured by the VL system have higher resolution and richer defect details. The high reflectivity of the VL system can easily lead to the annihilation of critical information in defect regions. The defect detection images of the IL system have high contrast and penetrating power, but their low resolution makes it difficult to obtain detailed information about the defects. Combined with the characteristics of each optical detection channel of the multi-sensor system, the proposed FMP method is utilized to register and fuse the defect detection images of visible and infrared light, which can effectively improve the richness of detection information and the defect detection ability under complex working conditions. The fusion-processed inspection image has a stronger ability to distinguish the detailed information of the defect area, which can effectively improve the contrast and clarity of the image and can highlight the edge contours of defects, such as pores, cracks, and scratches. The experimental results show that the image processed by the proposed FMP method is superior to other contrast algorithms in terms of the preservation and sharpness of the contrast and contour edge details in the target area, which makes the image rich in detail and more convenient for visual observation.

To evaluate the quality improvement effect of defect detection images objectively and quantitatively after the fusion of multi-sensor data, and to compare and analyze the detection images of visible and infrared light, the spatial frequency (*SF*), peak signal-to-noise ratio (*PSNR*), structural similarity (*SSIM*), average gradient (*AG*), edge intensity (*EI*), information entropy (*E*), and standard deviation (*SD*) [44,45,46] were used. Assuming that the size of the fused image is X×Y, the seven image performance evaluation indicators are as follows.

The *SF* can reflect the overall activity level of the image in the spatial domain. The larger the *SF* value, the better the quality of the fused image [44], defined as:

(17) $$\{\begin{array}{l} SF=\sqrt{CF^{2}+RF^{2}}\\ RF=\sqrt{\frac{1}{X\left(Y-1\right)}}\sum_{x=1}^{X}\sum_{y=2}^{Y}{\left(I_{x,y}-I_{x,y-1}\right)}^{2}\\ CF=\sqrt{\frac{1}{\left(Y-1\right)X}}\sum_{x=2}^{X}\sum_{y=1}^{Y}{\left(I_{x,y}-I_{x-1,y}\right)}^{2} \end{array}$$

where the SF represents the spatial frequency, CF is the spatial column frequency, and RF is the spatial row frequency.

The *PSNR* measures the similarity between two images from the gray level of the image. It can effectively reflect the statistical average value of the change of the image signal-to-noise ratio. It is the most used objective evaluation index of image quality. It is defined as:

(18) $$\{\begin{array}{l} PSNR(A,B)=10\log_{10}(\frac{255^{2}}{MSE\left(A,B\right)})\\ MSE(A,B)=\frac{1}{X\times Y}\sum_{i=1}^{X}\sum_{j=1}^{Y}\left[A\left(i,j\right)-B\left(i,j\right)\right] \end{array}$$

where A(i,j) and B(i,j) represent the grayscale of image *A* and image *B*, respectively, MSE(A,B) represent the mean square error between image *A* and image *B*, and the image size is X×Y. The unit of PSNR(A,B) is dB, and the larger the value, the smaller the deviation between image *A* and image *B*.

*SSIM* is an evaluation index that measures the similarity between two images, mainly including contrast, intensity, and structure. It is an objective evaluation index that is closer to subjective visual perception. The larger the structural similarity value, the higher the similarity between the two images, and the better the structural information is preserved [44], defined as:

(19) {SSIM(A,B)=(2μAμB+c1μA2+μB2+c1)α·(2σAσB+c2σA2+σB2+c2)β·(σAB+c3σAσB+c3)γμA=A¯=1N∑i=1NAi,μB=B¯=1N∑i=1NBiσA=[1N−1∑i=1N(Ai−μA)]12,σB=[1N−1∑i=1N(Bi−μB)]12σAB=∑i=1N(Ai−μA)(Bi−μB)

where ${\left(\frac{2\mu_{A}\mu_{B}+c_{1}}{\mu_{A}^{2}+\mu_{B}^{2}+c_{1}}\right)}^{\alpha }$ represents a similar degree of image brightness, ${\left(\frac{2\sigma_{A}\sigma_{B}+c_{2}}{\sigma_{A}^{2}+\sigma_{B}^{2}+c_{2}}\right)}^{\beta }$ represents a similar degree of image contrast, and ${\left(\frac{\sigma_{AB}+c_{3}}{\sigma_{A}\sigma_{B}+c_{3}}\right)}^{\gamma }$ represents the similarity degree of image structure.

$\mu_{A}$ and $\mu_{B}$ are the mean values of the brightness of image *A* and image *B*, respectively, and $\sigma_{A}$ and $\sigma_{B}$ are the standard of the brightness of image *A* and image *B*, respectively. $\sigma_{AB}$ is the covariance difference. α, β, and γ are the weight parameters to adjust the brightness, contrast, and structure terms, respectively. $c_{1}$, $c_{2}$ and $c_{3}$ are the constants used to ensure the balance of the formula.

The *AG*, also known as the grayscale, reflects the changes in the details and clarity of the image and is a measure of the image’s ability to express the contrast of details and texture information [46]. The AG is defined as:

(20) $$AG=\frac{1}{\left(X-1\right)\left(Y-1\right)}\sum_{i=0}^{X-1}\sum_{j=0}^{Y-1}\sqrt{\frac{I_{x}^{2}+I_{y}^{2}}{2}}$$

where $I_{x}=I\left(i+1,j\right)-I\left(i,j\right)$ represents the horizontal gradient information in the image (i,j), and $I_{y}=I\left(i,j+1\right)-I\left(i,j\right)$ represents the vertical gradient information in the image (i,j). 

The *EI* is essentially the magnitude of the image edge point gradient, that is, the local variation intensity of the image along the edge in the normal direction. The larger the edge strength value is, the more obvious the edge effect of the image is, which is of great significance in defect identification and extraction. For an image I(i,j), the Canny operator detects edges and the edge strength of the image at a point (i,j) is expressed as:

(21) $$\{\begin{array}{l} EI(i,j)=\sqrt{E_{i}^{2}+E_{j}^{2}}\\ E_{i}=\frac{\partial G}{\partial i}*I\left(i,j\right)\\ E_{j}=\frac{\partial G}{\partial j}*I\left(i,j\right)\\ G\left(i,j\right)=\frac{1}{2\pi \sigma^{2}}\exp(-\frac{i^{2}+j^{2}}{2\sigma^{2}}) \end{array}$$

where G(i,j) represents the center edge point operator, and $\frac{\partial G}{\partial i}$ and $\frac{\partial G}{\partial j}$ are the gradients of the graph in the *ij* direction, respectively. ∗ represents the convolution operation.

*E* is an index to measure the richness of image information. The larger the information entropy value, the greater the contrast of the image, the greater the amount of information, and the better the effect of image fusion. defined as:

(22) $$E=-\sum_{i=0}^{L-1}P_{i}\log_{2}\left(P_{i}\right)$$

where L represents the total gray level of the image, and $P_{i}$ is the proportion of pixels with the gray level i in the image to the total pixels.

The *SD* can reflect the grayscale difference information of the image, measure the difference between the source image and the fusion image, and compare and evaluate the fusion quality more intuitively. The *SD* is defined as:

(23) $$\{\begin{array}{l} SD=\sqrt{\frac{1}{XY}\sum_{i=0}^{X-1}\sum_{j=0}^{Y-1}{\left[I\left(i,j\right)-\bar{I}\right]}^{2}}\\ \bar{I}=\frac{1}{XY}\sum_{i=0}^{X-1}\sum_{j=0}^{Y-1}I\left(i,j\right) \end{array}$$

where $\bar{I}$ represents the mean value.

**Figure 7:**
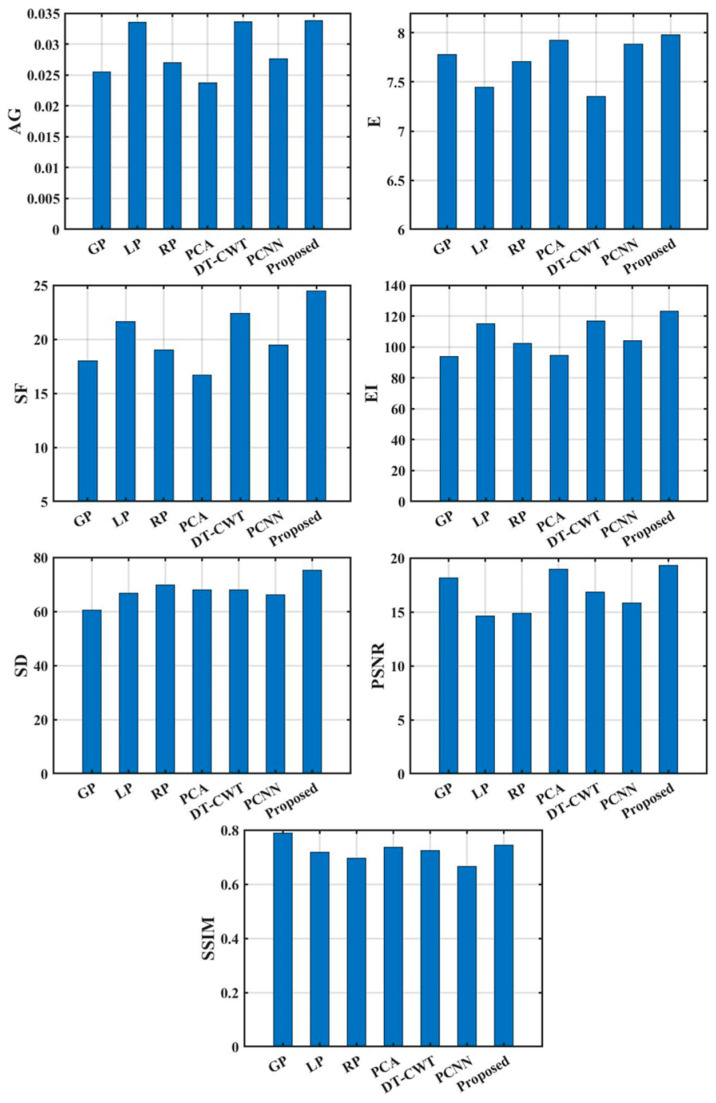
Comparison of objective evaluation indicators of dataset 1 under different fusion algorithms.

**Figure 8:**
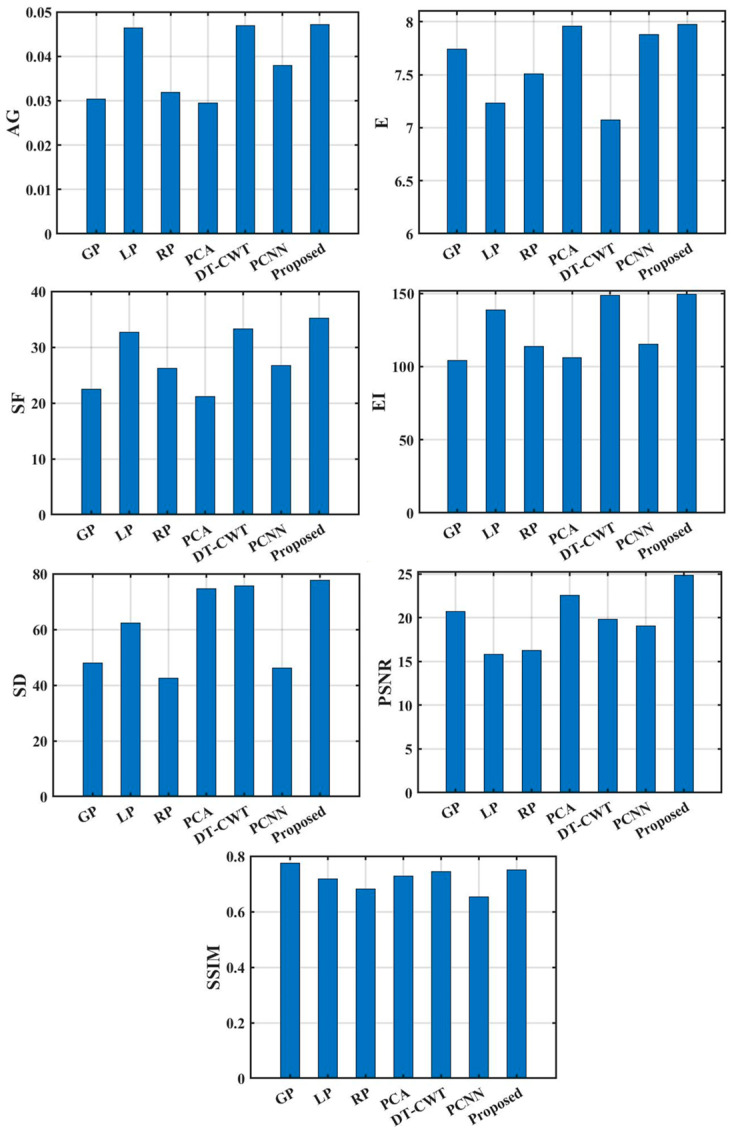
Comparison of objective evaluation indicators of dataset 2 under different fusion algorithms.

Figure 7 and Figure 8 are the objective evaluation index results obtained by the image fusion of dataset 1 and dataset 2 with the FMP method and seven comparison algorithms. The FMP method has obvious advantages in various indicators. From the image processing results of dataset 1, the *AG* of the fused image is 0.0338, the *E* is 7.983, the *SF* is 24.450, the *EI* is 123.327, the *SD* is 75.225, the *PSNR* is 19.325, and the *SSIM* is 0.745. From the image processing results of dataset 2, the *AG* of the fused image is 0.0472, the *E* is 7.975, the *SF* is 35.222, the *EI* is 149.635, and the *SD* is 77.683, the *PSNR* is 24.830, and the *SSIM* is 0.750. From the *AG* index, the average improvement rate of the FMP is 43.906% relative to the GP algorithm, 1.310% relative to the LP algorithm, and 36.574% relative to the RP algorithm. The average improvement rate of the PCA algorithm is 51.308%, which is 0.617% relative to the DT-CWT algorithm, and 23.501% relative to the PCNN algorithm. The images fused by the FMP have richer gradient information and a stronger ability to express the contrast of image details and texture information.

From the *E* index, the average improvement rate of the FMP is 2.836% compared with the GP algorithm, 8.717% compared with the LP algorithm, and 4.890% compared with the RP algorithm. The average improvement rate compared to the PCA algorithm is 0.446%, the average improvement rate compared to the DT-CWT algorithm is 10.664%, and the average improvement rate compared to the PCNN algorithm is 1.211%. The image contrast of the FMP is higher and more informative. From the perspective of the *SF* index, the average improvement rate of the FMP is 46.305% relative to the GP algorithm, 10.472% relative to the LP algorithm, and 31.564% relative to the RP algorithm. The average improvement rate of the PCA algorithm is 56.228%, which is 7.485% compared to the DT-CWT algorithm, and 28.813% compared to the PCNN algorithm. The image fusion performance of the FMP is better. Compared with the GP, LP, RP, PCA, DT-CWT, and PCNN algorithms, the average improvement rate of the FMP on the *EI* index is 37.399%, 7.472%, 25.929%, 35.691%,3.133%, and 24.088%, indicating that the FMP has more obvious image edge effects, which is conducive to the realization of defect recognition and feature extraction in the defect detection system. The average improvement rates on the *SD* indicators are 43.088%, 18.662%, 45.096%, 7.245%, 6.631% and 40.916%, respectively. The average improvement rates on the *PSNR* index are 13.101%, 44.522%, 41.313%, 5.992%, 19.937% and 26.093%, respectively.

From the *SSIM* index, compared with the LP, RP, PCA, DT-CWT, and PCNN algorithms, the average improvement rates of the FMP are 3.944%, 8.542%, 2.013%, 1.738%, and 13.271%, respectively. When compared with the GP algorithm, the SSIM index dropped by 4.417%, but within the acceptable range, and the overall quality effectively improved after image fusion processing.

The experimental results analysis above indicates that the FMP has significant advantages on the subjective and objective evaluation indicators. The FMP can effectively improve the image contrast and information richness, improve the display of image edge contour and texture information, and effectively retain and fuse the source image. Therefore, the proposed FMP method can significantly detect the defects of the PBF workpiece and carry out multi-sensor information fusion, and effectively analyze the defects after image processing. In addition, it provides a useful and potential solution for defect detection and processing parameter optimization in PBF processes. Furthermore, the design scheme of the FMP method is also applicable to other multi-sensor visual inspection systems, such as welding, laser cutting, and so on.

## 4. Conclusions

The quality of PBF parts may be seriously affected by certain defects (such as cracking and balling) during production and processing, resulting in poor quality and repeatability. In the process of using the multi-sensor detection system to detect and characterize the defects of the PBF parts, the quality of the detection images captured by the sensor is likely to be poor due to uncertain factors, such as changes in the detection environment, and it is difficult to analyze the detail features of defects. This paper designed a multi-source image acquisition system to simultaneously acquire brightness intensity and infrared intensity. Meanwhile, a multi-sensor image fusion method based on FDST-MSSTO and an improved PCNN framework (FMP) was proposed. Firstly, the principles of the FDST, MSSTO, and improved PCNN method are illustrated. Then, the FMP method was proposed, including the following procedures: the FDST is used to decompose the low-frequency sub-band coefficients and high-frequency sub-band coefficients of the source image, and the MSSTO is utilized to extract the bright information and dark information of image details in the low-frequency sub-band coefficients, the bright and dark information and low-frequency coefficients are fused to obtain low-frequency fusion coefficients, the improved PCNN method is used to obtain high-frequency fusion coefficients, the final fusion image is reconstructed by the inverse transform of the FDST. Meanwhile, the image fusion performance evaluation indicators, such as the averaged information entropy (*E*), average gradient (*AG*), spatial frequency (*SF*), standard deviation (*SD*), peak signal-to-noise ratio (*PSNR*), and structural similarity (*SSIM*) are illustrated. The experimental results show that the proposed FMP method achieves a satisfactory performance in terms of the *E*, *AG*, *SF*, *EI*, *PSNR*, and *SSIM*, which are 7.979, 0.0405, 29.836, 76.454, 20.078, and 0.748, respectively. Furthermore, the FMP is compared with the GP, LP, RP, PCA, DT-CWT, and PCNN algorithms. The experimental results show that the average improvement rates of the FMP method are 3.944%, 8.542%, 2.013%, 1.738%, and 13.271% when compared with the LP, RP, PCA, DT-CWT, and PCNN algorithms from the *PSNR* index. From the *SSIM* index, when compared with the LP, RP, PCA, DT-CWT, and PCNN algorithms, the average improvement rates of the FMP method are 3.944%, 8.542%, 2.013%, 1.738%, and 13.271%, respectively. Thus, the FMP method can effectively improve the image contrast and information richness, improve the display of image edge contour and texture information, and effectively retain and fuse the main information in the source image, which is of great significance for defect detection and processing parameter optimization in PBF processes. Furthermore, the design scheme of the FMP method can also be extended to other multi-sensor visual inspection systems, such as welding, laser cutting, etc.

## Figures and Tables

**Table 1:** Design parameters of the defect detection system.

Design Parameters	Visible System	Infrared System
Wavelength (μm)	0.4–0.7	0.9–1.7
Image sensor type	CMOS	InGaAs
pixel count	7728 × 5368	320 × 256
Pixel size (μm)	1.1	30
Focal length *f* (mm)	50	50
F-number	2.5	1.5
Object field size (mm)	51.90 × 36.30	58.50 × 46.98

## Data Availability

The data presented in this study are not publicly available at this time but may be obtained from the authors upon reasonable request.
